# The Epidemiology of Emergency Calls in a Tertiary Emergency Department for Admitted Patients: A TECOR Study

**DOI:** 10.3390/healthcare13141670

**Published:** 2025-07-10

**Authors:** Viet Tran, Toni Dunbabin, Simone Page, Lauren Thurlow, Giles Barrington

**Affiliations:** 1 Royal Hobart Hospital, Tasmanian Health Service, Hobart 7000, Australia; 2 Tasmanian School of Medicine, University of Tasmania, Hobart 7000, Australia; 3 Tasmanian Emergency Medicine Research Institute, Hobart 7000, Australia; 4 Menzies Institute for Medical Research, University of Tasmania, Hobart 7000, Australia

**Keywords:** medical emergency team, rapid response system, emergency department, goals of care, deteriorating patient

## Abstract

Emergency calls, including medical emergency team and code blue calls, aim to respond to and assess patients at an earlier stage of clinical deterioration (to potentially avoid cardiac arrest and death). With an increasing prevalence of hospital access block, more admitted patients are boarding in the Emergency Department (ED). Although emergency calls were traditionally a ward-based system, they are now occurring more often in the ED. Large variations exist in the staffing mix and specialist skill sets between ED- and ward-based care. There is a paucity of evidence describing the epidemiology of patients that require emergency calls in the ED setting. **Objectives:** We aim to evaluate the population of adult patients that require emergency calls in our tertiary ED. **Methods:** This study utilised the Tasmanian Emergency Care Outcomes registry (TECOR) to perform a retrospective cohort study of emergency calls occurring over a 13-month period. Descriptive statistics are used to summarize the data. Categorical variables are presented as frequencies and their percentages and continuous variables are depicted as means and standard deviations (SDs) or medians and interquartile ranges (IQRs), as appropriate. **Results:** There were 600 emergency calls in the ED, involving 423 unique patients and 596 (99.33%) MET calls. The mean patient age was 68.68 years (SD 17.87). The mean ED length of stay for patients with an emergency call was 18.28 h (SD 8.96). Calls made were predominantly for systolic blood pressure < 90 mmHg (310, 51.67%). The mean duration of an emergency call was 40.89 min (SD 20.48). Most patients had a single emergency call (311, 73.18%). For our cohort, goals of care remained unchanged following 341 (56.83%) emergency calls. **Conclusions:** Emergency calls in the ED are uncommon, representing 2.08% of all admissions from the ED. Patients in the ED requiring an emergency call have a mean length of stay twice that of all ED presentations. Recognising and responding early to address the concerns that trigger a call may help to mitigate this additional burden. More research is required to explore the factors that will lead to effective and appropriate care before, during, and after an ED emergency call is made.

## 1. Introduction

Research from the early 2000s indicated that around 10% of hospitalized patients experienced serious adverse events that were unrelated to their medical conditions, frequently linked to prior abnormalities in vital signs and leading to death or disability in 25% of patients [1,2,3,4,5]. This evidence prompted the development and implementation of rapid response systems (RRSs) made up of triggers that aim to detect and activate teams to assess patients at an earlier stage of clinical deterioration (e.g., those with clinical signs of impending cardiac, respiratory, or neurological failure) to potentially avoid cardiac arrest and death [6,7].

The utilization of an emergency call system is now widespread and incorporates objective clinical triggers to call for early assistance, in addition to subjective concerns [1,2]. This circumvents the influence and bias of human error that may otherwise prevent this escalation process from occurring [8]. Two systematic reviews have shown that the use of rapid response systems reduces the number of unintended cardiac arrests and all-cause mortality [9,10]. The exact underlying mechanisms through which emergency call implementation can enhance patient-centred outcomes is still unclear [11,12]. Australia was an early adopter of the RRS model, prompted by national policies to introduce recognition and response systems in all Australian hospitals [13].

Emergency departments around the world are seeing significant overcrowding [14]. This is where demand outstrips resources, diluting the quality of care able to be delivered and increasing the potential for harm [14]. A cornerstone of this harm is the ED boarding of patients, that is, patients who have been assessed, managed, referred, and admitted by an inpatient team but remain in the ED due to the lack of a bed on the ward, otherwise known as hospital access block [15]. It has been documented in the literature that increased lengths of stay in the ED are related to more adverse outcomes, including higher mortality and morbidity [14,16,17,18].

Rapid response teams have previously been unnecessary in the ED, as patients often present in a deteriorated state and are assessed and resuscitated from the outset. The clinical care provided is, thus, focused on resuscitation, stabilisation, and differentiation. Once this is achieved, referral and disposition are also key to ED management. The ED environment does not cater well to patients who require the routine and rigor of ward-based care at both a nursing and medical level. It has been reported that undetected clinical deterioration occurs in up to one in seven ED patients [19]. Given the worsening crisis of hospital access block, EDs are finding that emergency team activations are being called more often in the ED. Given the differences in staffing, supervision, and workflow between the ED and ward, how emergency call teams operate in this new setting, what patient populations are involved, what triggers such calls, and what outcomes are common are all relatively unknown.

Our primary objective is to evaluate the incidence of emergency calls in our ED. Our secondary objectives include evaluating the indications for an emergency call, demographic relationships, ED length of stay (LOS), diagnosis, and management during the review.

## 2. Materials and Methods

### 2.1. Setting

This study was conducted at a major tertiary mixed referral–teaching ED in Australia with an annual presentation rate of just over 75,000 patients, of which approximately 65% are adults based on local definition (14 years and older) [3]. Our ED is staffed by a range of doctors, including specialists, registrars, residents, and interns. The ED is divided into 5 distinct areas of care, including a resuscitation area for high-acuity patients, an acute area for patients requiring bed-based care, a low-acuity area, a paediatric area where patients require beds and closer nursing observation, and a short-stay unit. The ED also has an 8-bed area where predominantly admitted patients board.

The emergency call criteria used in our ED are the same as on the ward and consist of the following two-tier system: (1) code blue for cardiac arrest and/or imminent airway threat and (2) medical emergency team (MET) call for clinical deterioration (Table 1, Appendix A). Our health service is analogue-based, including nurse-led handwritten observation charts and manual counting for scores. There is variability in members responding to ED emergency calls in Australia [20]. Members who attend emergency calls in our ED include a medical registrar, a senior ED doctor, medical resident, medical intern, the in-charge ED nurse, and the ED nurse assigned to that patient’s care. In contrast to ward-based emergency calls, ICU staff do not attend ED emergency calls, but a referral can be made for consultation, including the consideration of transfer to the ICU.

### 2.2. Methods

Australia does not have a national data system. Our department has established its own ED clinical quality registry. This study utilised the Tasmanian Emergency Care Outcomes registry (TECOR) (ACTRN12624000278538) to perform a retrospective cohort study of emergency calls between 1 February 2024 and 28 February 2025 [16]. Data entry into TECOR for the emergency call module included a combination of automated entry for demographic details and other routinely collected ED data for federal reporting. The remaining data was manually entered. We sought to evaluate gender, age, the area of the ED where the call was made, Australian Triage Scale, ED length of stay, disposition, and the most common diagnosis based on ICD-10 coding.

### 2.3. Inclusion Criteria

The inclusion criteria included all patients presenting to the ED aged 14 years and over for the period analysed and those who had an emergency call (s) whilst in the ED. Medical emergency team calls were defined as activation of the emergency number through the switch board. The activation of MET calls may be prompted by physiological abnormalities in the patient, subjective concerns raised by staff members, or issues voiced by family members or visitors, in accordance with the hospital’s established policies and procedures for MET activation (Table 1).

### 2.4. Exclusion Criteria

The exclusion criteria included patients for whom there was no documented evidence of an emergency call taking place. Activations for non-admitted patients (e.g., outpatients), visitors, or staff outside of the ED were also excluded. Duplicate records from the TECOR were also excluded from analysis. Emergency calls for paediatric patients and those originating from patients in the short-stay unit were also excluded.

### 2.5. Data Analysis

Descriptive statistics are used to summarize the data. Categorical variables are presented as frequencies and their percentages and continuous variables are depicted as means and standard deviations (SDs) or medians and interquartile ranges (IQRs), as appropriate.

### 2.6. Ethical Approval

Ethical approval was provided by the Tasmania Health and Medical Human Research Ethics Committee (HREA30260, 26 February 2024).

## 3. Results

### 3.1. Incidence of Emergency Calls

During the study period, there were 83,238 ED presentations, 69,025 (82.92%) of which were for patients aged 14 years and over. Of these, 20,404 (29.56%) were admitted under an inpatient specialty team other than Emergency Medicine. There were 600 adult emergency calls made in the ED during this period, involving 423 unique patients (2.08% of all admissions).

### 3.2. Characteristics of Patients Requiring an Emergency Call

The mean age was 68.68 years (SD 17.87), with females representing 51.83% of all emergency calls made (Table 2). The acute area was the most common area of the ED where an emergency call was made (340, 56.67%), followed by the resuscitation room (238, 39.67%), with very few calls made from the subacute area (16, 2.67%) and minor injury area (6, 1.00%). Patients who presented with an Australian Triage Scale (ATS) score of two were featured most in the emergency calls made (266, 62.88%), followed by those with an ATS score of three (116, 27.42%). For emergency calls made, 369 (87.23%) were eventually transferred to the ward, while 46 (10.87%) were transferred to the intensive care unit (Table 2). Sepsis was the most common diagnosis for patients who had an emergency call during their ED stay, amounting to 78 patients (13.00%), followed by generally unwell/general illness not otherwise specified with 45 patients (7.50%).

**Figure 1:**
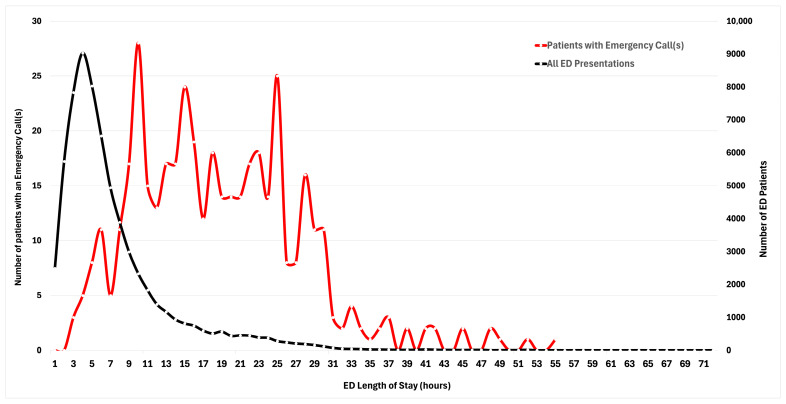
ED length of stay for all ED presentations versus ED patients with an emergency call.

The mean ED LOS was 6.77 h (SD 6.03) for all adult patients compared with 18.28 h (SD 8.96) for patients requiring an emergency call (Figure 1). When comparing ED LOS based on disposition, patients who were transferred to the intensive care unit had a shorter ED LOS (14.05 h) compared with those that were transferred to the ward (18.0 h).

### 3.3. Emergency Call Characteristics

For all emergency calls made, the majority were MET calls (596, 99.33%) compared with code blue calls (4, 0.67%) (Table 3). The most common reasons for an emergency call included systolic blood pressure < 90 mmHg (310, 51.67%), oxygen saturations < 90% (114, 19.00%), and heart rate > 140 beats per minute (84, 14.00%). Serious concern triggered 41 (6.83%) emergency calls. The mean duration of an emergency call was 40.89 min (SD 20.48). Most patients for whom an emergency call was made only had one call during their ED stay (309, 73.05%). However, 80 (18.91%) had a second emergency call during their ED stay, with 16 (3.78%) patients having a third call, 12 (2.84%) patients having a fourth call, and 3 (0.71%) patients having a fifth call (Table 3).

### 3.4. Emergency Call Management and Outcomes

Following attendance by the emergency response team, modifications to the Adult Deterioration Detection Score (ADDS) may be made to acknowledge the state of deterioration and recognize that it may be this way for some time. For patients who had an emergency call in the ED, the most common ADDS modifications were for systolic blood pressure (256, 42.67%), oxygen saturation (125, 20.83%), and heart rate (92, 15.33%) (Table 4).

The goals of care (GOCs) alpha numerical definitions for our hospital include A for cardiopulmonary resuscitation and all appropriate life-sustaining treatments; B not for cardiopulmonary resuscitation, may be for intubation; C not for cardiopulmonary resuscitation or intubation, may be for MET call; and D not for cardiopulmonary resuscitation or intubation and not for any emergency calls. Revisiting the goals of care for a patient is also common following an emergency call. For our cohort, 341 (56.83%) GOCs were unchanged, with 203 (33.83%) incomplete prior to the emergency call and 191 (31.83%) incomplete following the emergency call. For GOCs relating to ultimate disposition, ICU transfer occurred in 29 (4.83%) of GOC A, 1 (0.17%) of GOC B1, and 30 (5.00%) of GOC B2 patients (Table 5).

Ward-based patients who have an emergency call typically either remain on the ward for their acute management or are transferred to the intensive care unit if requiring a higher degree of care. The ED has the additional benefit of having different areas that can accommodate for higher care levels. For emergency calls in the ED, most patients remained in the area where a call was made (443, 77.17%), with 100 (16.67%) moving to the Resuscitation Room and 28 (4.67%) moved out of the ED to the ICU (28, 4.67%).

Delays in emergency calls, that is, when emergency call criteria were met but not called, were also recorded. For all emergency calls in the ED, 109 (18.17%) had a delayed call, with a mean delay of 86.07 min (SD 89.17). The stated reason for delay was absent in 44 cases (7.33%), with the most common reason being misidentification of severity in 37 (6.17%).

## 4. Discussion

### 4.1. Challenges of Emergency Department Emergency Calls

Emergency call systems play an important role in the changing landscape of EDs, where more patients now board awaiting inpatient beds [21]. There are obvious differences between the ward environment and ED, and emergency call systems need to adapt to these differences to be effective [22]. Another factor that must be considered when developing an emergency call system specific for the ED is to understand the types, triggers, and dispositions of this cohort of patients to improve their care. Our study describes, for the first time in the literature, the epidemiology of emergency calls for admitted adult patients in an ED setting, occurring almost twice per day on average [23]. Similar studies around emergency calls are inconsistent with regard to the patient population being evaluated for emergency call responses. Therefore, drawing comparisons can be difficult [23].

### 4.2. Characteristics of ED Emergency Calls

We found that the mean age for our cohort was 69 years, marginally higher when compared with ward-based studies, where age typically ranges from 63 to 77 years [7,24].

The most common trigger for emergency calls in our cohort was SBP < 90 (310, 51.67%), with the next most common being O_2_ < 90% (114, 19%) [24]. This is of some concern, since it has been well described that, of all the common triggers for an emergency call, low blood pressure is a relatively late sign [25,26]. Only 41 (6.83%) were called for serious concern. This may relate to emergency department staff unofficially reviewing patients to reassure concerns or support an escalation of care. In one study of MET calls within 24 h of admission from the ED, it was found that triggers included a decreased level of consciousness (19.4%), hypotension (17.5%), and clinical concern (13.5%) as the three most common causes [24]. In another study of MET calls in orthopaedic patients, the most common triggers were hypotension (37.5%), tachycardia (25.0%), and tachypnoea (9.1%) [27].

Our study also found that there were 109 (18.17%) delays to MET calls, with the most stated reason being due to missed severity of illness (37, 6.17%). Delayed activation of emergency calls within our cohort appeared to be less when compared to the ward literature, where 17–29% were delayed [28,29,30].

The mean duration of an emergency call was 40.89 min. We were unable to find a comparator in the literature [23]. This should be considered when managing the governance of a rapid response system, as this is a significant amount of clinician time for a team to commit to day-to-day, as a clinician might have other duties to manage.

Quite often, it is the lack of clear understanding of a patient’s wishes before they deteriorate that prompts discussions of what care may be considered futile or in their best interest, which is subsequently documented in GOCs. This is a challenging task, made even more so with relatives now in a state of distress given the situation. Concerningly, 203 patients (33.83%) who had an emergency call did not have GOCs documented prior to the call. Even more concerning was that 191 patients (31.83%) did not have one following an emergency call, which raises concerns given that this population is the mostly likely to deteriorate. We suspect that one justification for this is due to the treating team not always being present for the emergency call response, especially if it occurs out of hours, and, therefore, these conversations are deferred until the treating team is available.

We note that within the cohort evaluated, 1 patient had eight emergency calls, with 114 patients (26.95%) having two or more emergency calls. There is a paucity of evidence evaluating the frequency of emergency calls for individual patients on the ward for comparison. The reason for multiple emergency calls for individual patients in the ED may include the absence of expertise from ICU staff within the responding emergency call teams servicing the ED, as well as access block to the ICU. Another reason may be the result of ED shift work patterns, where boarded patients are handed over multiple times during their ED stay. This lack of continuity, together with the safety risks that are well described with handover, may potentially contribute to both lower-quality care and an increased frequency of emergency calls [31].

### 4.3. ED Length of Stay

The median ED LOS for all patients who had an emergency call in the ED was 17.4 h (IQR 11.20–24.10). When comparing patients who were transferred to the ward versus the ICU, the median LOS was 18.0 vs. 14.05 h. The shorter ED LOS for those that were transferred to the ICU was likely due to a higher degree of bed availability in the ICU compared to the ward. Patients that require ICU-level care in the ED likely take up limited resources such as resuscitation bays and, therefore, are prioritised over those that are not a burden on the ED, given the potential for critically unwell patients to present to the ED. When comparing ED length of stay for all ED presentations versus ED patients with an emergency call (Figure 1), it is evident that patients who had an emergency call spent longer in the ED. One reason for this finding is that patients underwent a prolonged resuscitation in the ED to be deemed vitally safe and align their care needs with ward expectations. Another reason may be due to the often-protracted nature of establishing GOCs with patients and/or relatives where the presentation and deterioration are often unexpected and distressing.

### 4.4. Disposition of Emergency Call Patients in ED

Ward-based emergency calls are challenged by a disposition dichotomy—patients either remain on the ward or are considered too unwell and are transferred to the intensive care unit. Delays to transfer reportedly incur an increase in mortality by 1.5% for each hour of delay [32]. The ED, on the other hand, is structured to receive undifferentiated patients across the spectrum of acuity [33]. Therefore, it has well-defined areas to manage patients with similar needs, ranging from a resuscitation room for the critically unwell to the minor injuries area for lower-acuity patients. This enables more efficient resource stewardship [16]. The disposition of patients following an emergency call is, therefore, more varied, with options for patients to remain in their clinical area, move to a high-acuity area of the ED (the resuscitation bay), or be transferred to the ICU. This contrasts with ward-based emergency calls, where a wide range has been reported for unplanned ICU admissions, from 5.4% to 68.81% [24,34,35,36,37]. Our study found that only 46 patients (10.87%) were ultimately transferred to the ICU. This low number of patients transferred to the ICU from the ED also supports the theory that there is no other area in the hospital to support high-acuity patients after their initial resuscitation and differentiation in the ED. The increased mean LOS of 18.28 h compared with all ED presentations of 6.77 h further supports the theory that the degree of vital sign derangement sits outside the scope and comfort of ward clinicians, but is not deranged enough to be eligible for admission to the ICU. A prolonged LOS in the ED, therefore, raises additional concerns given that new undifferentiated patients who are potentially critically unwell will continue to present and may compete for the limited number of resuscitation bays available [38]. Furthermore, there is nuance in the management of patients by medical and nursing staff, with emergency staff, intensive care staff, and ward staff all having specific skill sets and expertise. Although emergency staff are well trained in initial stabilisation, differentiation, and resuscitation, expertise begins to drift the longer that patient is in the ED, with evidence showing that the longer patients board in the ED, the higher their mortality and morbidity [14].

### 4.5. Emergency Call Characteristics Unique to ED

Most patients had their emergency call activation in the acute area of the ED (340, 56.67%), with only 100 of all non-resuscitation-room-activated calls moved to the resuscitation room following the emergency call review. The second-highest emergency call activations occurred in the resuscitation room (238, 39.97%), which is of concern given that the role of the resuscitation room in the ED is to resuscitate and stabilise patients. An emergency call in this area means that the time course for improvement may be prolonged or first- and second-line interventions are not working as effectively as required. These patients, therefore, require a higher level of care than a ward can provide in terms of medical expertise, review frequency, and, more importantly, nursing care. We postulate that a significant number of MET calls occurred in the resuscitation bay because of ongoing high care needs that are not available on the ward. This is supported by the outcomes for MET calls, where 429 calls (71.50%) had ADDS modification to prevent another MET call, suggesting that patients were still being actively resuscitated with high care needs. This contrasts with reports in the literature for ward-based emergency calls, where only 6.0% had modifications [39]. The reason for this is likely temporal, with patients likely having ADDS modifications during their initial care in the ED and prior to arriving on the ward.

Australian EDs utilize the Australian Triage Scale to categorise patients in order of urgency from one, being most critical, to five, being least. Most patients in our study who activated a MET call had ATS scores of two and three (266, 62.88%, and 116, 27.42%). The high number of ATS 2 calls was likely due to the extended time often needed to resuscitate and stabilize critically ill patients. The large number of ATS 3 calls is surprising, and identifying this is important to ensure that patients do not suffer an adverse outcome because of clinician complacency due to triage bias, that is, the cognitive biases that can affect decision making during the triage process, leading to potential errors in patient prioritization and outcomes [40]. Our study found that there were only 20 calls (4.73%) for ATS 1 patients, which is unsurprising, as these patients are typically critically ill and require urgent specialist procedural intervention such as surgery or angiography and meet the threshold for admission to the intensive care unit due to their criticality.

### 4.6. Future Directions for ED MET Call Research

With the unrelenting growth of ED overcrowding due to access block, the prevalence of clinical deterioration in admitted patients boarding in the ED will also grow. Our research shows that this scenario is both a burden on ED staff and a safety risk for patients. The earlier detection and mitigation of deterioration in the ED is a growing area of research, particularly with some promising insights around machine learning and artificial intelligence able to predict deterioration in patients yet to decompensate [41].

The management of critically unwell patients also varies between EDs, with studies on septic shock stabilising the patient in the ED at some hospitals, transferring them immediately to the ICU, or having an ED-based ICU [42,43,44,45].

As previously described, the MET team that attends the ED does not include an ICU medical practitioner or an ICU nurse. Although some studies have shown a significant improvement in patient outcomes for patients who are cared for by intensive care clinicians whilst awaiting transfer to the ICU, there is limited data to for the broader cohort of patients who may need ward-based care [32,46,47].

Finally, ward-based emergency calls have evolved, with some now introducing pre-MET review to respond earlier in the trajectory of deterioration [48].

### 4.7. Limitations

This was a registry analysis of observational data, and, therefore, interpretation is limited to this methodology. Observational data, while valuable for generating hypotheses and identifying associations, is inherently limited by potential confounding variables, selection bias, and the inability to establish causality.

Our data collection and data analysis limited the ability to compare in-hospital mortality and hospital length of stay, and we hope to address this in future iterations of the project.

We also treated each MET call separately, and, therefore, patients who had more than one MET call were over-represented in the population characteristics.

## 5. Conclusions

Patients who require an emergency call in the ED represent a burden for the service and face risks with longer lengths of stay. Patient populations were similar to those found for ward-based emergency calls, however, there were differences in the triggers for a call, with fewer calls occurring due to clinician concern, likely a result of the proximity of and access to medical staff in the ED. Alarmingly, patients with emergency calls in the ED had a mean LOS of 18.28 h compared with the mean ED LOS for all presentations of 6.77 h. Recognising and responding early to address the concerns that trigger a call may help to mitigate this additional burden. More research is required to explore the factors that will lead to effective and appropriate care before, during, and after an ED emergency call is made.

## Figures and Tables

**Table 1:** Adult emergency call criteria.

Call Type	Criteria
MET Call	Any observation in the purple area ^1^
	A total ADDS ^2^ score greater than or equal to 8 ^1^
	Airway threat
	Serious concern about the patient
	New drop in oxygen saturation < 90%
	Sudden fall in level of consciousness
	Seizure
Code Blue	Impending or actual cardiac or respiratory arrest
	Impending or actual airway compromise

^1^ Colour and numerical chart available in Appendix A. ^2^ ADDS—Adult Deterioration Detection Score.

**Table 2:** Population characteristics of patients who had an emergency call in the ED.

Category	Variable	n (%)
Gender	Female	222 (52.48)
	Male	201 (487.52)
	Total	423
Age	Mean (years ± SD)	68.68 (17.87)
	Range (min–max)	19–102
	Median (IQR)	72 (58–82)
ED Area	Resuscitation Room	238 (39.67)
	Acute Area	340 (56.67)
	Minor Injury Area	6 (1.00)
	Subacute Area	16 (2.67)
Australian Triage Scale	1	20 (4.73)
	2	266 (62.88)
	3	116 (27.42)
	4	17 (4.02)
	5	-
	8 ^1^	4 (0.95)
ED Length of Stay	Mean (hours ± SD)	18.28 (8.96)
	Range (min–max)	2–54.3
	Median (IQR)	17.4 (11.20–24.10)
Disposition, n (%), Mean LOS (hours)	ICU	46 (10.87), 14.05
	Ward	369 (87.23), 18.00
	Usual Place of Residence	4 (0.95), 11.20
	Other Hospital	2 (0.47), 22.85
	Died in ED	2 (0.47), 22.65
Top-Ten ED Diagnosis (ICD-10), n (%)	Sepsis (A41.9)	78 (13.00)
	Generally Unwell/General Illness NOS ^2^ (R69.0)	45 (7.50)
	Pneumonia, NOS (J18.9)	24 (4.00)
	Bacterial Pneumonia, NOS (J15.9)	22 (3.67)
	COPD, Acute Exacerbation NOS ^1^ (J44.1)	20 (3.33)
	Atrial Fibrillation or Atrial Flutter (I48.9)	18 (3.00)
	Delirium (F05.9)	16 (2.67)
	Intestinal/Bowel Obstruction (K56.6)	15 (2.50)
	Congestive Heart Failure (I50.0)	12 (2.00)
	Chronic Obstructive Pulmonary Disease (J44.9)	12 (2.00)

^1^ Australian Triage Scale 8 not officially recognized but a local designation to reflect a patient returning to the ED from the short-stay unit and ^2^ Not Otherwise Specified.

**Table 3:** Emergency call characteristics.

Category	Variable	*n* (%)
Call Type	MET Call	596 (99.33)
	Code Blue	4 (0.67)
Call Reason	Airway Threat	4 (0.67)
	Difficulty Breathing	14 (2.33)
	Respiratory Rate < 4	0 (0.00)
	Respiratory Rate > 35	24 (4.00)
	Oxygen Saturation < 90%	114 (19.00)
	Heart Rate < 40	16 (2.67)
	Heart Rate > 140	84 (14.00)
	Systolic Blood Pressure < 90	310 (51.67)
	Glasgow Coma Scale < 10	59 (9.83)
	Seizure	9 (1.50)
	Serious Concern	41 (6.83)
	ADDS ^1^	2 (0.33)
	Other	4 (0.67)
Call Duration	Mean (minutes ± SD)	40.89 (20.48)
	Range (min–max)	5–198
	First and Third Quartile (IQR)	30, 46
Number of Emergency Calls	1	309 (73.05)
	2	80 (18.91)
	3	16 (3.78)
	4	12 (2.84)
	5	3 (0.71)
	6	2 (0.47)
	7	-
	8	1 (0.24)

^1^ ADDS—Adult Deterioration Detection Score.

**Table 4:** Emergency call management and outcomes.

Category	Variable	*n* (%)
ADD ^1^ Modifications	None	171 (28.50)
	Systolic Blood Pressure	256 (42.67)
	Heart Rate	92 (15.33)
	Oxygen Saturation	125 (20.83)
	Oxygen Flow Rate	0 (0)
	Temp	0 (0)
	Consciousness	17 (2.83)
	Other	2 (0.33)
Goals of Care	Unchanged	341 (56.83)
	A to B ^2^	2 (0.33)
	A or B to C ^2^	16 (2.66)
	A or B or C to D ^2^	12 (2.00)
	Incomplete Before Emergency Call	203 (33.83)
	Incomplete After Emergency Call	191 (31.83)
Disposition	Unchanged	443 (77.17)
	Moved to Resuscitation Room	100 (16.67)
	Moved to ICU	28 (4.67)
	Died	1 (0.17)
	Moved Other	8 (1.33)
Delays to MET	Delay to MET	109 (18.17)
	Mean (minutes ± SD)	86.07 (89.17)
	Range (min–max)	10–686
	First and Third Quartile (IQR)	38, 103
Reason for Delay(s)	None Stated	44 (7.33)
	Appropriate Staff in Attendance	0 (0)
	Message Sent	28 (4.67)
	Called Incorrect Level of Clinician	20 (3.33)
	Called ED Clinical Review	4 (0.67)
	Severity Not Identified	37 (6.17)

^1^ Adult Acute Deterioration Scale and ^2^ Goals of Care alpha numerical definitions, A for cardiopulmonary resuscitation and all appropriate life-sustaining treatments; B not for cardiopulmonary resuscitation, may be for intubation; C not for cardiopulmonary resuscitation or intubation, may be for MET call; and D not for cardiopulmonary resuscitation or intubation and not for any emergency calls.

**Table 5:** Goals of Care on discharge from ED following an emergency call.

Disposition n (%)	A	B1	B2	C1	C2	D	No GOC	Total
Died in ED	0	0	0	0	0	2 (0.33)	0	2 (0.33)
ICU	29 (4.83)	1 (0.17)	30 (5.00)	0	0	0	19 (3.17)	79 (13.17)
Other Hospital	0	0	3 (0.50)	0	0	0	2 (0.33)	5 (0.83)
Usual Place of Residence	2 (0.33)	0	0	0	0	0	2 (0.33)	4 (0.67)
Ward	115 (19.17)	12 (2.00)	172 (28.67)	18 (3.00)	12 (2.00)	13 (2.17)	168 (28.00)	510 (85.00)
Total	146 (24.33)	13 (2.17)	205 (34.17)	18 (3.00)	12 (2.00)	15 (2.50)	191 (31.83)	600 (100.00)

Goals of Care alpha numerical definitions; A for cardiopulmonary resuscitation and all appropriate life-sustaining treatments; B not for cardiopulmonary resuscitation, B1 for intubation, B2 not for intubation; C not for cardiopulmonary resuscitation or intubation, C1 for MET call and C2 for non-MET call; and D not for cardiopulmonary resuscitation or intubation and not for any emergency calls.

**Figure A1:**
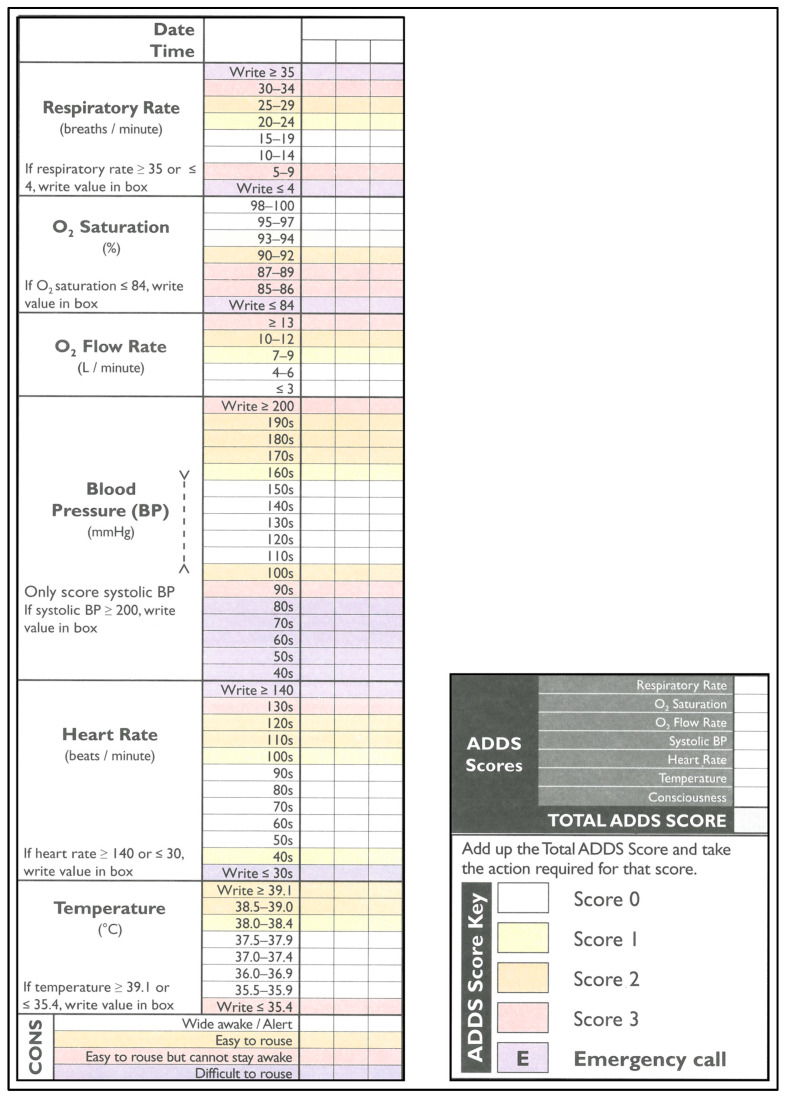
Adult Deterioration Detection System Chart.

## Data Availability

The data presented in this study are available on request from the corresponding author due to local privacy laws.
